# Ab Initio Manganese K*α* and K*β* Energy Eigenvalues, Shake-Off Probabilities, Auger Rates, with Convergence Tests

**DOI:** 10.3390/molecules29174199

**Published:** 2024-09-04

**Authors:** Jonathan William Dean, Scott Neil Thompson, Christopher Thomas Chantler

**Affiliations:** School of Physics, University of Melbourne, Parkville 3010, Australia; jonathan.dean@unimelb.edu.au (J.W.D.); snthompson@student.unimelb.edu.au (S.N.T.)

**Keywords:** X-ray fluorescence, MCDHF, relativistic quantum mechanics

## Abstract

This work presents ab initio calculations for the Kα spectrum of manganese (Z = 25, [Ar]$3d^{5}4s^{2}$), a highly complex system due to the five open orbitals in the 3d shell. The spectrum is composed of the canonical diagram line [1s]→[2p] and shake-off satellite lines [1snl]→[2pnl] (nl∈{2s,2p,3s,3p,3d,4s}), where square brackets denote a hole state. The multiconfiguration Dirac–Hartree–Fock method with the active set approach provides the initial and final atomic wavefunctions. Results are presented as energy eigenvalue spectra for the diagram and satellite transitions. The calculated wavefunctions include over one hundred million configuration state functions and over 280,000 independent transition energies for the seven sets of spectra considered. Shake-off probabilities and Auger transition rates determine satellite intensities. The number of configuration state functions ensures highly-converged wavefunctions. Several measures of convergence demonstrate convergence in the calculated parameters. We obtain convergence of the transition energies in all eight transitions to within 0.06 eV and shake-off probabilities to within 4.5%.

## 1. Introduction

X-ray fluorescence is widely used in science and industry due to its utility in a range of areas including non-destructive structural analysis, elemental abundance, environmental monitoring, and chemical composition. First-principles calculations of atomic spectra are useful as they may complement and test experimental data; are quick and cheap compared to experiments; have no Gaussian broadening, which allows portability to any detector resolution; can test state-of-the-art atomic physics; and, when performed on highly charged ions, offer tests of quantum electrodynamics (QED). The theoretical model for atom–electron and electron–electron interactions, and QED, has been tested in certain circumstances to incredible precision [1]. Important questions emerge when ab initio results do not agree with empirical data [2]. This is the case with certain X-ray spectra, especially the K-series fluorescence spectra within the 3d transition metals, where asymmetries, anomalous intensity ratios, and energy shifts are observed when attempting to fit theory to experiment [3,4,5]. Recent work on scandium Kα and Kβ [6,7] has shown the potential for great consistency between theory and experiment. Similarly, recent research on the copper K$\alpha_{3,4}$ satellite [8] holds the promise of recreating X-ray spectra from first principles.

K-series radiation is produced when an initial perturbation creates a core 1s vacancy that is filled by a 2p electron for Kα, and by a 3p electron for Kβ. Often, transitions are referred to in terms of their hole states with the use of square brackets, e.g., [1s]→[2p] for Kα. Spin–orbit interactions result in fine-structure, which is seen in the Kα profile of two well-resolved peaks: K$\alpha_{1}$ for $\left[1s\right]\to \left[2p_{3/2}\right]$ and K$\alpha_{2}$ for $\left[1s\right]\to \left[2p_{1/2}\right]$. Kβ X-rays are the result of the [1s]→[3p] transition and, as relativistic spin effects are reduced for higher orbitals, the $\left[1s\right]\to \left[3p_{3/2}\right]$ and $\left[1s\right]\to \left[3p_{1/2}\right]$ peaks are not well-resolved for the 3d transition metals.

Experimental data for the 3d transition metals show asymmetric peaks, satellite spectra, one-sided tails, and anomalous intensity ratios [4,5,9,10,11,12]. These features are not well described by the diagram, or canonical transitions, which result from the [1s]→[2p] or [1s]→[3p] [4,5,6,7,13,14]. Therefore, different atomic and solid-state phenomena have been hypothesised to account for these discrepancies including the radiative and non-radiative Auger effect, the Coster–Kronig effect, other decay channels, and shake-off and shake-up events. Recent work has shown that shake-off events are necessary but not sufficient to recreate expected spectral profiles for several transition metals [6,7,9,12,13,14,15,16,17].

Shake-off events, first proposed in [18,19,20,21], are when the initial perturbation creates a secondary ionisation in some nl subshell. If this is immediately followed by [1snl]→[2pnl] transition, with the Kα transition taking place with an nl spectator vacancy resulting in an altered potential, the resulting photon is an nl shake-off satellite photon. Similarly, for Kβ, the shake-off satellite lines are non-degenerate to the main, diagram spectra, since the transition takes place with an nl spectator vacancy, which alters the potential. Shake-off events greatly increase the complexity of calculations due to the inclusion of more electron-hole states. This work presents the Mn Kα and Kβ transitions for the diagram and all n∈{2,3,4} satellite lines.

Shake-off event probabilities are often calculated in the sudden, adiabatic, limit [22,23] and are often used to model shake-off satellite intensities directly. Recent work by Melia et al. [8] has shown that an nl shake-off event only leads to an nl shake-off satellite if the [1snl]→[2pnl] transition takes place *before* the nl hole is filled by some other mechanism. Therefore, this work also determines the Auger rates for these initial hole states to calculate the *Auger suppression factor* and to result in an improved shake-off satellite intensity. The Auger suppression is strongest for inner-shell electron shake-off events where many Auger decay channels exist. Hence, the impact of the Auger suppression on Sc Kα and Kβ [7] was minor.

Currently, 3d transition elements are of particular interest within atomic physics literature. Their open *d* orbital leads to complex electron structures, and more generally, the 3d transition metals have useful properties such as their magnetism, potential alloying for high-temperature superconductivity, and catalysts in chemical processes. The manganese system in its canonical ground state has the greatest number, five, of unpaired 3d electrons with the electron configuration [Ar]$3d^{5}4s^{2}$. Unpaired electrons add to the complexity of calculations by increasing the number of spin-coupling states and configuration states that must be considered. The complexities can be seen in over $2.8\times 10^{5}$ independent transition energies for the transitions and over $10^{8}$ configuration state functions required for well-converged wavefunctions.

This work presents a priori calculations of Kα and Kβ transitions. The first results presented are the energy eigenvalue spectra for the Kα and Kβ (in parentheses) diagram transitions [2p(3p)]→[1s], six nl shake-off satellite eigenvalue spectra [2p(3p)nl]→[1snl] for nl∈{2s,2p,3s,3p,3d,4s}, and the valence-to-core [3d]→[1s] transitions. As the energy eigenvalue spectra are presented, they are compared with previous literature. Following these, the ab initio shake-off probabilities are presented as percentages. Finally, first-principles Auger electron decay rate calculations determine the Auger effect corrected by ab initio shake-off satellite intensities.

The calculations are performed using the multiconfiguration Dirac–Hartree–Fock (MCDHF) method with the active set approach. The active set approach increases the convergence of wavefunctions. Several measures of convergence are discussed and presented.

The theoretical calculations are performed using the General Relativistic Atomic Structure Package, 2018 (GRASP-2018) suite of programs [24,25,26]. Our additional implemented software for GRASP improves the relativistic configuration interaction and QED effects [27,28] and is incorporated with GRASP-2018 [29]. The results are the eigenvalue spectra for the diagram and several satellite transitions as well as the shake-off probabilities. Auger transition rates are calculated using RATIP [30]. The calculations are performed with the University of Melbourne Spartan High-Performance-Computing system.

## 2. Prior Literature

Peng et al. [31] provide calculations of the Mn Kβ spectrum in three oxidation states—Mn (II), Mn (III), and Mn (IV)—using nonrelativistic wavefunctions. The shape of their calculated spectrum and the experimental data suggest undisclosed energy shifts; so, it is hard to determine the accuracy of their eigenenergies. The shape differs significantly between oxidation states, they do not calculate a neutral Mn Kβ profile, and they do not provide their empirical energy shifts; thus, no meaningful comparison can be made between [31] and the ab initio Kβ from this current work.

Jonnard et al. [15] perform nonrelativistic calculations for both Kα and Kβ spectra, specifically aimed at the I(Kα):I(Kβ) intensity ratio. Similar to Peng et al. [31], the spin states are fixed and populated statistically, resulting in fewer and simpler computations. Jonnard et al. [15] perform calculations to test three different ground states for the Mn atom with the 3d subshell populated by four, five, or six electrons, with the 4s subshell remaining empty. There is some difference between the three ground state electron configurations. Our calculations are performed for a neutral atom with the canonical Mn ground state electron configuration [Ar]$3d^{5}4s^{2}$. Comparisons of our work with [15] are possible, but the different initial state quantum systems will play a significant part in any comparison, as discussed elsewhere [32,33].

Deslattes et al. [34] provide an extensive tabulation of theoretically derived emission energies for isolated atoms, including Mn K$\alpha_{1}$, K$\alpha_{2}$, K$\beta_{1}$, K$\beta_{2}$, K$\beta_{3}$, and K$\beta_{5}$. This work performs calculations in a relativistic framework, hence improving on the previous works. However, this work only presents a single energy value for each spectrum, apparently given by the diagram line only at the single configuration level (initial 4s level of expansion only), preventing comparison between the theoretical and experimental spectral shapes. Moreover, the Kβ transitions are given individually, whereas in all experimental spectra the transitions overlap, resulting in a single peak for K$\beta_{1,3}$ and K$\beta_{2,5}$. Nonetheless, we are able to compare [34] with the mean energy of our eigenenergy (diagram) spectra.

Mitra et al. [35] perform nonrelativistic calculations for the Kα and Kβ transition in Mn as well as for some high-energy satellite lines. In Kα, they consider an L shell spectator vacancy, an L shell double spectator vacancy, and a K shell spectator vacancy, resulting in a hollow atom and a hypersatellite. In Kβ, they only consider the L shell single spectator vacancy. The authors of [35] include the full set of spin states resulting in many eigenvalues and, from this, many transition energies. Unfortunately, they do not report eigenvalue spectra and only report a single weighted mean energy. Mitra et al. [35] use the theoretical calculations to fit to experimental data over a wide energy range—over 1 keV. The experimental resolution is not great enough to resolve individual spectral shape. Hence, we are unable to compare theoretical spectral profiles and can only compare absolute energies.

Diamant et al. [36] perform calculations on the Mn $K^{h}$$\alpha_{1,2}$ hypersatellite, with calculations provided in a relativistic framework and the full eigenvalue spectrum reported. However, that work only presents the hypersatellites.

We also compare our theoretical results with experimental data. As such, it is essential to include detector resolution and theoretical broadening. This current work presents three individual metrics for the central tendency of energy: the peak energy eigenvalue $E^{\infty }$ or the peak of a spectrum collected with infinite resolution; the peak of a reconstructed profile with resolution given by literature (experimental) full-width at half-maximum (FWHM) $E^{0}$; and the centre of mass or weighted mean energy $E^{\mathrm{CoM}}$, the peak energy for a detector with no resolution.

## 3. Transition Energies

The Hartree–Fock method is a self-consistent field approach to solving many-electron wavefunctions [37,38]. The Dirac prefix indicates the replacement of the nonrelativistic Schrödinger wave equation with the relativistic, four-component, Dirac equation. The manganese canonical ground state is $\left[Ar\right]3d^{5}4s^{2}$, which has the maximal number of unpaired 3d electrons, each of which can take total angular momentum values $j\in \{\frac{3}{2},\frac{5}{2}\}$. These angular momenta couple, along with the initial hole in the 1s shell, such that the total angular momentum can take values j∈{0,1,2,3,4,5,6,7}. After the Kα transition, the hole in the 2p shell leads to the possibility of j=8. With shake-off events comes an extra hole in the electron configuration, leading to further angular momentum coupling and an even larger set of angular momenta. The method of calculation herein follows Dean et al. [7] on scandium. This work, however, consists of a far greater number of possible spin states resulting in a large number of eigenvalues and, therefore, transition energies.

Wavefunctions are obtained for the initial [1s] and [1snl] states and the final [2p] and [2pnl] states for each nl shake-off satellite. Solutions to the MCDHF equations are given through the variational approach and obtaining the wavefunction energies is performed with the extended optimal level (EOL) energy functional [24]. Each initial and final wavefunction is optimised individually, and transition energies are calculated once biorthogonalisation of the wavefunctions has been performed.

As observed in recent theoretical work using single configuration Dirac–Hartree–Fock (DHF) calculations [39,40,41], the ground state configuration state function (CSF) energy eigenfunctions are insufficiently accurate for well-resolved spectra. The single configuration does not allow for major electron–electron correlations, which change the energy eigenvalues by up to 2 eV for the 3*d* transition metals. The electron–electron correlations are accounted for by defining an active set of CSFs that includes virtual orbitals above the ground state and allowing a certain number of electrons to excite into the active set from the ground state. For this work, we allow two electrons to excite into the virtual orbitals. The set of orbitals from which the electrons may be excited from, and the virtual orbitals they may be excited to, is defined as the *active set* . Once the active set is expanded beyond the highest occupied ground state orbital, the term *multiconfiguration* is applied to the DHF method—hence, MCDHF.

**Figure 1:**
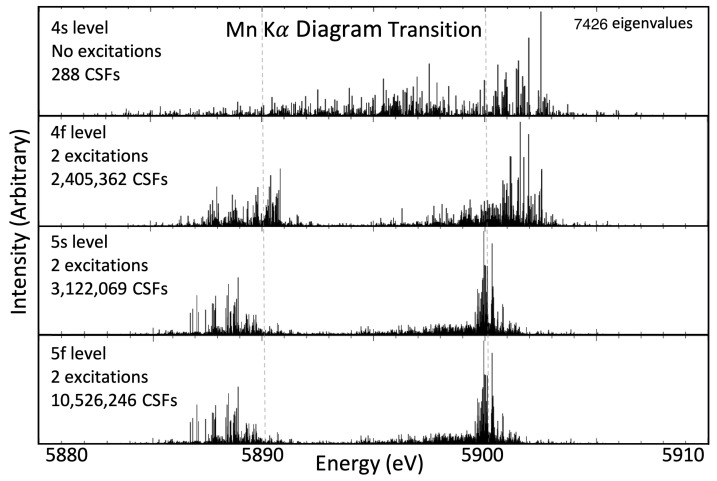
The energy eigenvalues for the Mn Kα diagram transition. Each successive panel shows an expansion of the active set. The improvement of the result is clear from the 4s to 4f and then 5s shells being included in the active set, and the increased active set from 5s to 5f shows good convergence of results. The number of CSFs required at each step is given, with over 10 million for the 5f level, and there are 7426 total eigenvalues.

The active set approach is an iterative method where initial calculations are performed in the single configuration and include an active set of all occupied orbitals {1s,…,4s}. The atomic wavefunction calculated from the single configuration approach is then used as an initial estimate for the wavefunction that includes virtual orbitals up to the 4p level. An active set is defined with the *frozen core approximation*, where, for Kα, the n∈{1,2} orbitals are no longer part of the active set, and for Kβ, the frozen core extends to include the 3s and 3p orbitals. Furthermore, during the calculations of an nl shake-off satellite wavefunction, the particular nl orbital is also removed from the active set. The list of CSFs increases rapidly as every combination of electron configuration is allowed, from choosing zero, one, or two electrons from the active set orbitals and allowing them to exist in the list of active virtual orbitals. Unlike the single configuration calculation at the 4s level, only the 4p orbital is optimised at this level. The next level, the 4d level of calculation, just adds the 4d orbitals to the active set, and optimisation is only performed on this orbital. This process repeats to the 5f orbital. The necessity of the active set approach can be observed in Figure 1, which presents the energy eigenvalue spectrum for the Kα diagram transitions. The first panel shows the results with the active set expanded to the 4s orbital (single-configuration, no excitations); the second, third, and fourth panels show the eigenvalue spectra with the active set expanded to 4f, 5s, and 5f, respectively (multiconfiguration, two allowed excitations). In Figure 1, there is a noticeable difference between the energy eigenvalue spectra calculated from panel one to panel two, and again from panel two to three. Successfully, no readily discernible difference is apparent between panels three and four. Observing the qualitative convergence gives some confidence that the wavefunctions have converged at the 5f level of expansion. Quantitative measures must be defined and are presented for all transitions (Section 11). Notice that the number of independent transition energies is rigorously defined by the quantum mechanical coupling of open shell spin and angular momentum states.

Herein, the energy eigenvalue spectra for all considered transitions are all presented at the 5f level of expansion of the active set in the Supplementary Information. Comparisons to previous theoretical literature values and experimental values are also presented.

## 4. K$\alpha_{1,2}$ Near-Degenerate Satellites

**Figure 2:**
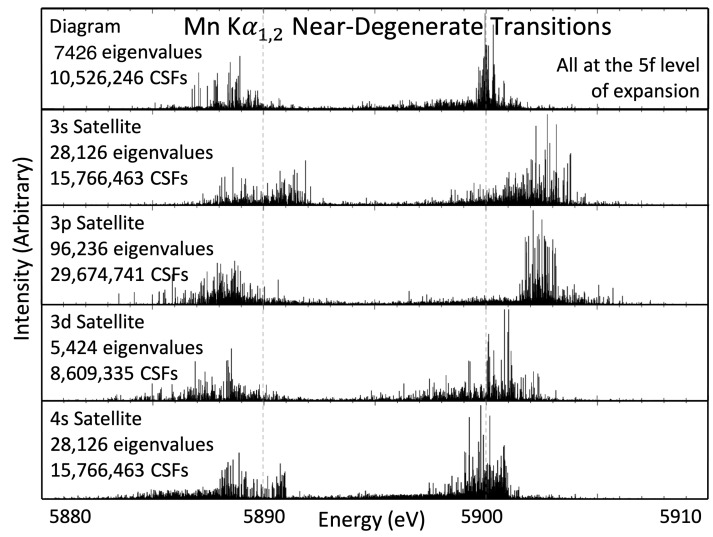
Eigenvalue spectra for the transitions that compose the Mn K$\alpha_{1,2}$ spectrum. The eigenvalue height represents the intensity $g_{f}$ relative to other eigenvalues in the same set of transitions—that is, within the same panel of the figure—and not between different sets of transitions. The diagram transitions dominate and are the largest contributor to the measured spectra. Relative intensities for the shake-off satellites and diagram are presented in Table 9. Measures of convergence of these eigenvalue spectra are presented in Section 11.

The eigenvalue spectra for the diagram transition and near-degenerate nl shake-off satellites for the Mn K$\alpha_{1,2}$ profile are presented in Figure 2. The transition energy height $g_{f}$ represents the intensity relative to the other transition energies in the same set. The intensities $g_{f}$ are calculated *given that* the particular $nl\to n^{\prime }l^{\prime }$ electron transition is taking place and, as such, do not relate to fluorescent yield or Auger decay. Each of the sets of transitions gives an obvious K$\alpha_{1,2}$ structure—that is, they are easily discernible into $2p_{1/2}$ and $2p_{3/2}$ subsets. The K$\alpha^{\prime \prime }$ satellite appears as a high-energy shoulder on the K$\alpha_{1}$ peak [7,12]. A leading hypothesis is that it is due to the 3p shake-off satellite. This work supports that hypothesis, with the most intense, peak eigenvalue for the 3p satellite spectrum just over 2 eV greater than the peak of the diagram line. The K$\alpha_{2}$ peak in the 3p shake-off satellite has a similar energy to the diagram line, suggesting that the relativistic fine-structure splitting effect is greater in the 3p shake-off satellite by roughly 2 eV. The 3s shake-off satellite line is also roughly 2 eV greater than the diagram line. However, due to its relatively low intensity, it is not a major contributor to the K$\alpha^{\prime \prime }$ feature. The 3d and 4s shake-off satellites are highly degenerate with the diagram line, which supports the findings in work on scandium and copper [6,13,14].

Several previous studies offer comparison with the results of this work. Other theoretical work include Deslattes et al. [34] and Jonnard et al. [15]. Past empirical works are more extensive (Table 1) [4,15,21,42,43,44,45]. The values for Bearden [43] were presented in units of ${Å}^{*}$, which have since been corrected for and are presented in Hölzer et al. [42]. We use the corrected results. The values of Parratt [21] were presented in X units and have been converted to eV using the 2006 CODATA values for the fundamental constants [46]. Where possible, uncertainties are given, which are taken from the published results or given from our own digitisation of their plots.

One issue arises since there are several distinct ways to report an energy, depending on the definition of central tendency, in both theoretical and empirical studies. Dean et al. [11] provides an analysis of these different centroid definitions with an emphasis on experimental characteristic X-ray spectra. From a list of a priori derived eigenvalues, the most intense may be chosen $E^{\infty }$, where the infinity denotes a spectrum of infinite resolution. Especially, for a system with as many energy transition energies as manganese $E^{\infty }$ is a naïve measure. A better estimate is the average over all *N* transition energies to give a centre of mass (CoM) energy $E^{\mathrm{CoM}}$—specifically, $E(K\alpha_{1}^{CoM})$. Here, the CoM is calculated by weighting the *n*-th energy eigenvalue by its intensity $g_{f}$:

(1) $$E^{CoM}=\frac{\sum^{N}\left(E_{n}g_{fn}\right)}{\sum^{N}g_{fn}}$$

A preferred third method requires a width to be defined for the transitions; then, a reconstructed profile is determined a priori, and the peak of this spectrum may be taken as the energy value $E^{0}$ or K$\alpha_{1}^{0}$. We believe the empirical studies report the peak of the spectral lines $E^{0}$; yet, often with a largely unknown broadening width or profile point spread function. The theoretical work of Jonnard et al. [15] presents the peak of the simulated spectrum $E^{0}$. We presume that Deslattes et al. [34] reports the diagram line only at the single configuration level (initial 4s level of expansion only).

These measures are presented in Table 1. For the $E^{0}$ energy, an FWHM is required to recreate a spectral peak, for which the values from Hölzer et al. [42] are used, which is also assumed to be Lorentzian in profile: 2.47 eV (K$\alpha_{1}$), 2.92 eV (K$\alpha_{2}$), and 2.97 eV (K$\beta_{1,3}$). The shake-off satellite spectra add further complexities. The shake-off satellite spectra are an intrinsic part of the empirical measurements. We recreate the spectra including the shake-off satellites with the same widths [42], with the relative intensities we present in Table 9. Table 1 presents the three previous literature values and our results for the three measures of central tendency.

Most empirical X-ray spectra have energies presented that are the peak intensity of the spectrum. Therefore, these should compare best with $E^{0}$. A simple average of the previous experimental work yields $E(K\alpha_{1})=5898.90$ eV and $E(K\alpha_{2})=5888.06$ eV. Relative to these, our theoretical estimates for $E^{0}$ are $\Delta E_{K\alpha 1}^{0}=0.691$ eV and $\Delta E_{K\alpha 2}^{0}=0.535$ eV, marking an improvement over the previous theoretical calculations. Rather than comparing a single energy metric, state-of-the-art theoretical calculations should be able to recreate the full structure of an X-ray profile with its asymmetries. Tran et al. [45] recently measured high-precision Mn Kα spectra at the Diamond Light Source synchrotron, resolving structure with high resolution. These theoretical calculations compare well with the empirical data, considering the structure with both diagram and satellite spectra.

## 5. K$\alpha_{3,4}$ Transitions

**Figure 3:**
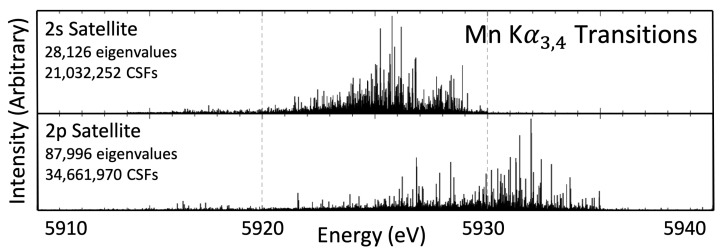
The n=2 shake-off satellite transitions that result in the Mn K$\alpha_{3,4}$ high-energy satellite. These high-energy satellites are roughly 30 eV above the K$\alpha_{1,2}$ spectrum. The fine-structure double-peak is no longer easily visible in these transitions. Similar to Figure 2, the height of the eigenvalue represents its relative intensity within the set, not between sets of transitions, and the relative intensity between the 2s and 2p shake-off satellites is presented in Table 10.

The K$\alpha_{3,4}$ satellite is a high-energy satellite roughly 30 eV greater than the main K$\alpha_{1,2}$ profile for manganese. Figure 3 presents the two sets of transitions that are considered responsible for this satellite, namely, the 2s and 2p shake-off satellites. Table 2 presents the measures of central tendency for the K$\alpha_{3,4}$ satellite. Some investigations report the values of K$\alpha_{3}$ and K$\alpha_{4}$ separately [21], with the origin of the double peak resulting from fine-structure splitting in the 2p shake-off satellite transitions and being greatly reduced compared with the splitting in the K$\alpha_{1,2}$ spectrum. The 2s shake-off satellite could contribute to the splitting; however, its intensity is roughly 3% of the 2p shake-off satellite and will not dominate. For the reported values of K$\alpha_{3}^{peak}$ and K$\alpha_{4}^{peak}$, the 2p shake-off transition energies are used and arise from using the transition energies resulting from either $\left[2p_{3/2}2p\right]\to \left[1s2p\right]$ for $\alpha_{3}$ or $\left[2p_{1/2}2p\right]\to \left[1s2p\right]$ for $\alpha_{4}$. The values for $E^{\mathrm{CoM}}$ and $E^{0}$ are more challenging to report, and come from combining the two sets of transition energies weighted according to the ab initio intensities. For $E^{0}$, an FWHM is needed to reconstruct a theoretical profile, for which 1.82 eV is used following Tran et al. [45].

The fine-structure double-peak is not easily discernible for these satellites. The weak structure has been noticed in scandium [7], which also has a large number of transition energies (>100). The loss of structure means that it is not clear which transition energies are the result of a $2p_{1/2}\to 1s$ transition and which are the result of a $2p_{3/2}\to 1s$ transition. By contrast, copper found that the separated components are observed [8], noting that copper has far fewer energy eigenvalues. Similar to previous work on both copper and scandium, the 2p satellite has higher energies than 2s [7,8].

Fewer references are available for Mn K$\alpha_{3,4}$ than for Mn K$\alpha_{1,2}$ (Table 2). For ab initio calculations, the presence of an extra hole state leads to complexities, which explains, in part, the fewer theoretical investigations. Mitra et al. [35] also uses the MCDHF method and reports a K$\alpha_{3,4}$ energy of 5390.1 eV, consistent with ours. The K$\alpha_{3,4}$ satellite has a much smaller intensity than the main K$\alpha_{1,2}$, making observation difficult. Unlike K$\alpha_{1,2}$, the two peaks are not well resolved, meaning that some authors have reported the K$\alpha_{3}$ and K$\alpha_{4}$ peaks separately while others report the combined K$\alpha_{3,4}$ peak. The experimental data [21,35] are 2–3 eV removed from the theoretical values obtained in this work. The previously mentioned synchrotron work from Tran et al. [45] has also resulted in fits of these theoretical results to the empirical data for the K$\alpha_{3,4}$ hidden satellite in Sier et al. [48]. From this, there is strong evidence to support the results in this work as they fit the data very well, to $\chi_{r}^{2}=1.96$.

## 6. Mn K$\beta_{1,3}$ Transitions

**Figure 4:**
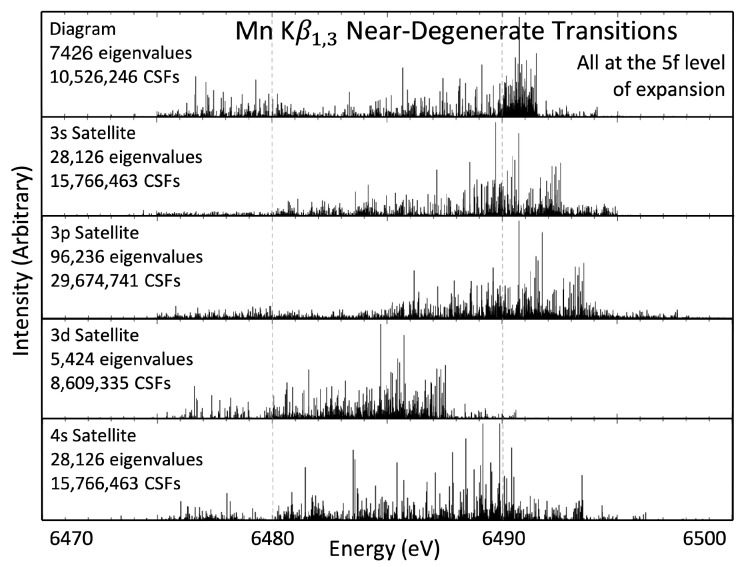
Eigenvalue spectra for the transitions that compose the Mn K$\beta_{1,3}$ spectrum.

The Mn K$\beta_{1,3}$ diagram and near-degenerate nl shake-off satellites are presented in Figure 4, analogously to Figure 2 and Figure 3. Fine-structure splitting is a relativistic effect and, therefore, decreases in magnitude as the principal quantum number increases; hence, the two transitions $\left[3p_{1/2}\right]$ and $\left[3p_{3/2}\right]\to \left[1s\right]$ are not well-resolved. Yet, it is still possible to present separate values for the K$\beta_{1}$ ($\left[3p_{3/2}\right]\to \left[1s\right]$) and K$\beta_{3}$ ($\left[3p_{1/2}\right]\to \left[1s\right]$) sets of transition energies, as seen in Table 3. Table 3 presents the results of the K$\beta_{1}$, K$\beta_{3}$, and full K$\beta_{1,3}$ eigenvalue spectra centroids with the three definitions explained earlier, peak of spectrum $E^{0}$, peak eigenvalue $E^{\infty }$, and CoM $E^{\mathrm{CoM}}$.

The three shake-off satellites—3s,3p, and 4s—are each highly degenerate with the diagram transitions. The 3d shake-off satellite shows an interesting energy shift, roughly 5 eV lower than the other sets of transitions. Some work has observed that an extra 3d electron hole leads to a negative shift to the 3d shake-off satellite energy [7]. However, the effect is much more dramatic here. This deserves further work, especially with fits to empirical data, since the 3d shake-off transition should be observable in well-resolved experimental spectra.

Comparisons between the results from this work and previous theory and experiment are presented in Table 3. The work from Deslattes et al. [34] and Jonnard et al. [15] that gave K$\alpha_{1,2}$ theory results also give K$\beta_{1,3}$ results, and are presented along with Mitra et al. [35], which did not give K$\alpha_{1,2}$ results. Deslattes et al. [34] do not give results for the K$\beta_{1,3}$, but rather the individual K$\beta_{1}$ and K$\beta_{3}$ spectra, whereas Jonnard et al. [15] and Mitra et al. [35] give the result for the combined, overlapping, K$\beta_{1,3}$ spectrum. There is a large discrepancy in the previous theoretical results, with a 17 eV difference between Jonnard et al. [15] and Mitra et al. [35]. Comparing these two with Deslattes et al. [34] is difficult since the latter give individual K$\beta_{1}$ and K$\beta_{3}$ peaks and the former give the combined K$\beta_{1,3}$ peak; however, as K$\beta_{1,3}$ lies between individual K$\beta_{1}$ and K$\beta_{3}$ peaks, we can say that Jonnard et al. [15] is at least 9.7 eV greater than Deslattes et al. [34]. Mitra et al. [35] reports a K$\beta_{1,3}$ peak, which is only 0.01 eV different to the Deslattes et al. [34] K$\beta_{3}$ value. Realistically, the K$\beta_{1,3}$ value should be much closer to the K$\beta_{1}$ value than K$\beta_{3}$, showing that there is a discrepancy in these values too. The K$\beta_{1}$ values from this work and Deslattes et al. [34] compare better, with each definition of centroid energy being within 3 eV of one another. There is a significant 7 eV difference between the K$\beta_{3}$ values.

The empirical data are far less spread, with all K$\beta_{1,3}$ energies lying within 0.82 eV of one another. To these, our report of spectral peak energy, K$\beta_{1,3}^{0}$, of 6490.701 eV compares extremely well with the empirical data, which have an average of 6490.472 eV over the five experiments. Further, the individual K$\beta_{1}$ and K$\beta_{3}$ peaks of Ito et al. [5], the only experimental work that presents these values, compare favourably again, with a K$\beta_{1}$ energy difference of 0.633 eV and a difference in K$\beta_{3}$ of 0.768 eV.

Theoretical calculations should not just be tested against its ability to compare with peak energy values but also on its ability to predict the shape of spectra. Recent synchrotron experiments have been conducted with high-resolution data, to which these theoretical values have been fit, which will be presented in a future paper.

## 7. K$\beta_{2,5}$ Transitions

**Figure 5:**
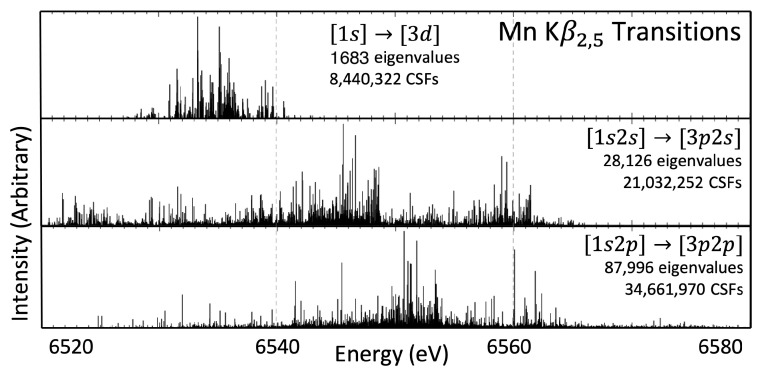
Eigenvalue spectra for the transitions that compose the Mn K$\beta_{2,5}$ spectrum.

The K$\beta_{2,5}$ satellite spectrum is the most interesting and complicated of the spectra considered herein. Initially, a satellite feature in the high-energy tail of the main K$\beta_{1,3}$ spectrum resembles the K$\alpha_{3,4}$ satellite, and one might assume the genesis is the same. However, Kβ transitions involve the n=3 shell, and the suppressed, electric quadrupole (Δl=2)[1s]→[3d] exists as a high-energy satellite as well. This suppressed transition is often referred to as a valence-to-core transition. The energy eigenvalue spectra from the valence-to-core transition and the 2s and 2p shake-off satellites are presented in Figure 5. The spectra overlap provides an interesting question over the origin of the Mn K$\beta_{2,5}$ satellite spectrum. Recent synchrotron experiments have taken high-quality data on the high-energy tail of the Kβ spectrum in order to test the relative intensity of the two origins of the K$\beta_{2,5}$ spectrum.

No theoretical results exist for the Mn Kβn=2 shake-off satellite. Mitra et al. [35] and Deslattes et al. [34] provide highly discrepant results for the [1s]→[3d] transition, with a difference of 11.36 eV. The values from this work lie closer to those of Deslattes et al. [34], yet with a large discrepancy of 5.356 eV and over 15 eV from those of Mitra et al. [35].

There exist three previous experimental values. Two are in agreement with Jonnard et al. [15] and Ito et al. [5], differing by 1.56 eV, with the value from Mitra et al. [35] being roughly 15 eV greater. Our value for the peak spectral energy $E^{0}$ compares reasonably with Ito et al. [5], yet with a difference of 1.372 eV. The difference between the value from this work and Jonnard et al. [15] is 2.928 eV. Mitra et al. [35] is very close to the reported Kβ2s and 2p shake-off satellites from this work, with differences of less than 1 eV. The similarities are most likely a coincidence.

Tran et al. [45] and Sier et al. [48] are interesting because they probe the evolution of the Mn K$\alpha_{3,4}$ satellite feature as a function of incident energy. Similar work is being conducted on the K$\beta_{2,5}$ feature, which will shed light on the origins of the spectra, since the relative intensity of the valence-to-core transition [1s]→[3d] and the n=2 shake-off transition [1s2l]→[3p2l] is a function of incident energy. The results from this work along with previous values for comparison are presented in Table 4.

## 8. Shake-Off Probabilities

As mentioned, the height of the energy eigenvalue in each of the previously presented spectra represents the probability of that particular transition *within* a set of transitions. In order to construct a fully theoretical X-ray spectrum, the relative intensity *between* each set of transitions must be found. The relative intensity between the diagram and each nl shake-off satellite are found from calculating the shake-off probability—that is, the probability that the system finds itself in either the diagram [1s] initial state or a potential nl shake-off satellite initial state [1snl].

Let $\varphi_{i}\left(N\right)$ be the *N*-electron eigenfunction of the ground-state atomic Hamiltonian *H*. If the ejection of the initial 1s electron occurs immediately and without interaction with the other electrons, then the new eigenfunctions $\varphi_{i}\left(N\left[1s\right]\right)$ are the same as before ionisation but with the loss of a 1s electron. This is the *sudden* or *adiabatic* approximation. After ionisation, the new Hamiltonian will be some $H^{\prime }$, and $\varphi_{i}\left(N\left[1s\right]\right)$ are not eigenfunctions of this post-ionisation Hamiltonian. Therefore, the wavefunction will relax into some new eigenfunction $\varphi_{i}\left(N\left[1snl\right]\right)$, now an eigenfunction of the new Hamiltonian $H^{\prime }$. During this relaxation process, there is a non-zero probability that a second electron, represented by nl in $\varphi_{i}\left(N\left[1snl\right]\right)$, is ejected into the continuum. The initial states can be represented as an expansion over the new states:

(2) $$|\varphi_{i}\left(N\right)\rangle =\sum_{j}c_{ij}^{2}|\varphi_{j}^{\prime }\left(N\left[1snl\right]\right)\rangle$$

where $c_{ij}^{2}={\langle \varphi_{j}^{\prime }\left(N\left[1snl\right]\right)|\varphi_{i}\left(N\right)\rangle }^{2}$ represents the probability for the system in an initial state $\varphi_{i}$ to be in a new state $\varphi_{j}^{\prime }\left(N\left[1snl\right]\right)$ after the sudden change of the Hamiltonian. For example, a 3p shake-off event is represented by $\varphi_{j}^{\prime }\left(N\left[1s3p\right]\right)$, and ${\langle \varphi_{j}^{\prime }\left(N\left[1s3p\right]\right)|\varphi_{i}\left(N\right)\rangle }^{2}$ represents the probability that a 3p shake-off has occurred. The full probability Pr(nl) can only be calculated when all decay channels are found and then normalised. The adiabatic limit is a good approximation as long as the perturbing energy is high enough; typically, the rubric of least three times the edge energy is considered, which is the case for all experimental characteristic reference spectra.

In order to recreate a spectral profile from ab initio calculations, two things are required. The energy eigenvalue spectra, as presented above, are necessary, as are the relative intensity of competing transitions. For the transitions presented, the shake-off satellites are the competing transitions to the diagram transitions. The nl shake-off probabilities, as calculated in Equation (2), are presented in Table 5. The values calculated here are compared with those from Mukoyama and Taniguchi [22], and Kochur et al. [23]. The shake-off probabilities are presented such that the sum of nl shake-offs and the diagram, i.e., no shake-off, sum to unity.

This work presents consistently greater shake-off probabilities than Mukoyama and Taniguchi [22], and lower probabilities than Kochur et al. [23], except for the 4s shake-off. These results are consistent with the previous two results for ab initio shake-off probabilities in Sc [7] and Cu [14]. For each shake-off probability, except the 3p and 3d, there are significant discrepancies. The previous work was limited by available computing power, which may have prevented good convergence of wavefunctions, especially for a system with as many open shells as manganese. Mukoyama and Taniguchi [22] solved nonrelativistic Hartree–Fock equations and then applied a relativistic correction, which may be the source of some of the discrepancy. Kochur et al. [23] are not clear on how they calculate their wavefunctions, so it is harder to speculate over the causes for any difference. Furthermore, Kochur et al. do not provide calculations for individual elements but instead presented a formula with sets of coefficients in order to obtain the shake-off probabilities. The ab initio nl shake-off probabilities give a naïve approximation for an nl shake-off satellite intensity, as a satellite photon is only observed as long as the Kα or Kβ, [1s]→[2p] or [1s]→[3p], transition takes place *before* the nl hole is filled by some other means. The most likely method for filling the hole is from the Auger effect. Therefore, to obtain a priori shake-off satellite intensities, the Auger, nonradiative rates and photonic, radiative rates must be found.

## 9. Auger Decay Rates

Shake-off probabilities alone are insufficient in obtaining shake-off satellite intensities. A shake-off event will only lead to the shake-off satellite being observed if the [1s]→[2p] transition takes place *before* the shake-off shell is filled. Therefore, the rates of other decay channels must be known if the true ab initio shake-off satellite intensities are to be determined. The Auger process is considered herein to represent the nonradiative decay channels.

Auger rates are calculated through the relativistic atomic transition and ionisation properties (RATIP) software package [30], designed to be used in conjunction with GRASP. The initial and final wavefunctions are found with the MCDHF approach using GRASP; then, RATIP calculates Auger properties such as kinetic energies, rates, and angular distribution parameters. The Auger rates between an initial bound state $\psi_{i}$, with energy *E*, and a set of final continua {$\psi_{f},\epsilon$} for electron kinetic energy ϵ are calculated following Fermi’s Golden Rule [49].

The rates calculated for the Auger decays permit calculations of the Auger suppression factor for the shake-off satellites. The Auger suppression factor represents the likelihood that a shake-off event leads to a shake-off satellite in the X-ray spectrum or decays first by a nonradiative path. One can treat the Auger suppression factor as a correction to the probability distribution of the X-ray spectrum. Therefore, the Auger suppression factor for a particular set of transitions, or nl shake-off, is valid only in its comparison to the other transitions in the spectrum being considered. For example, if the K$\alpha_{3,4}$ satellite has been isolated, then only the 2s and 2p shake-off satellites need to be considered [8].

This work considers all possible decay channels and gives rates in atomic units of eV/ℏ for the initial hole states [1s] and [1snl] for nl∈{2s,2p} in Table 6 and nl∈{3s,3p,3d,4s} in Table 7. These rates are then used to calculate Auger suppression factors for each of the shake-off satellites; then, the shake-off satellite intensities, along with the diagram intensity, are renormalised and presented in Table 8.

## 10. Satellite Intensities

Shake-off probabilities have been used to predict the shake-off satellite intensities in previous Kα studies [7,8,9,13,50]. This has had reasonable success for the K$\alpha_{1,2}$ spectral region. However, for the high-energy K$\alpha_{3,4}$ satellite, the 2s to 2p shake-off satellite intensity ratio I(2s):I(2p) has always obtained a theoretical prediction, suggesting a I(2s) far greater than observed. Indeed, if left as a free parameter in least-squares fitting, often I(2s) = 0 [8,50].

**Figure 6:**
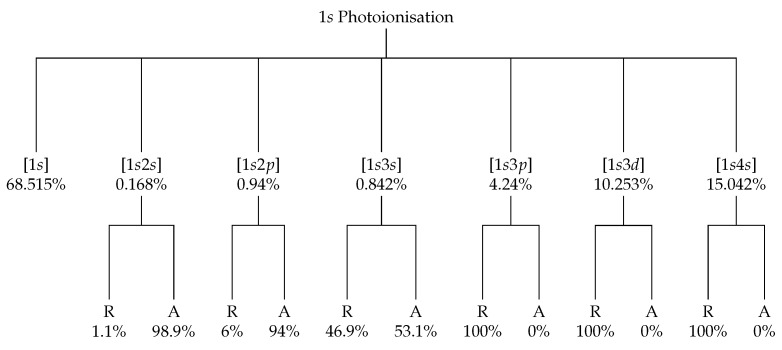
A tree diagram representing the possibilities after an initial 1s photoionisation. The hole states have the values from Table 5, which are the probabilities that an initial ionisation event, assuming the sudden approximation, will lead to the particular hole state. Then, the normalised probabilities for each hole state leading to an Auger (A) or radiative Kα photon (R) relaxation is given, with values at two significant figures, derived from Table 6 and Table 7.

The issue of the suppressed I(2s) was resolved for copper K$\alpha_{3,4}$ by including the Auger rates in an Auger suppression factor [8]. This work presents the Auger and radiative rates in Table 6 and Table 7, which are used to modify the shake-off probabilities from Table 5 to obtain the satellite intensities. The tree diagram in Figure 6 demonstrates why the Auger suppression is necessary and is especially important in K$\alpha_{3,4}$.

The first row of the tree diagram in Figure 6 are the probabilities that a 1s photoionisation leads to the particular hole state. These values are the shake-off probabilities from Table 5. The second row of the tree are the probabilities that the particular hole state leads to a radiative or nonradiative Auger emission, taken from Table 6 and Table 7. Since the satellite intensities involve observing X-ray photons resulting from the shake-off events, the intensities are calculated from the probability of obtaining a particular radiative decay and then renormalised. These intensities are presented in Table 8 for the full Kα spectrum. As many studies focus on either the K$\alpha_{1,2}$ or K$\alpha_{3,4}$ spectrum in isolation, the intensities for the relevant shake-off satellites renormalised for these spectra alone are given in Table 9 for K$\alpha_{1,2}$ and Table 10 for K$\alpha_{3,4}$.

We do not present the [1s] hole state rates as, to first-order, these are not important to the Auger suppression of satellite lines. The [1s] hole state does lead to Auger decay. However, the rate at which this occurs is within 1–2% of the rate that a [1snl] double ionisation decays via the Auger effect filling the 1s orbital—that is, the decay channels that are created as a result of the nl shake-off still contain the original Auger decay into the 1s orbital and at a rate that is comparable to the 1s Auger decay.

The shake-off probabilities and Auger rates are identical for Kα and Kβ calculations. However, there is a different rate for the radiative transitions, leading to different Auger suppression factors and satellite intensities. The Kβ intensities are presented in Table 11. No renormalised intensities for the isolated K$\beta_{1,3}$ profile are presented since the n=2 shake-off intensities are so small and the Auger suppression of them is so severe; thus, the probabilities do not change between the full Kβ or the isolated K$\beta_{1,3}$ up to the third decimal place. Further, unlike for K$\alpha_{3,4}$, we do not present the the K$\beta_{2,5}$ renormalised intensities due to the confusion of the valence-to-core transition versus the shake-off satellite origin of this spectral feature.

Since the rate of a Kβ transition is roughly seven times smaller than the Kα transition, the effect of Auger suppression is greater on Kβ. This is clearly seen when comparing the entries for the intensity of the 2s, 2p, and 3s shake-off satellites between Kα, Table 8, and Kβ, Table 11. The magnitude of the Kβ2p shake-off satellite relative to the full Kβ profile is roughly a factor of six smaller than the magnitude of the Kα2p shake-off relative to the Kα. Since the Kβ profile is already roughly a factor of seven times smaller than the Kα, the Kβn=2 shake-off satellites are roughly a factor of forty-two smaller than the Kαn=2 shake-off satellites that result in the K$\alpha_{3,4}$ spectrum. Therefore, it is most likely that all K$\beta_{2,5}$ spectra in the literature refer to the valence-to-core transition, which is not influenced by an Auger suppression.

The need for an Auger suppression factor in better modelling X-ray spectra for the K$\alpha_{3,4}$ satellite for 3d transition metals has been demonstrated with copper [8]. The impact of the Auger suppression on the K$\alpha_{1,2}$ spectrum is minimal due to few Auger decay channels being available for 3d transition metals with an n=3 vacancy [7,8]. Recent data collected on the Mn K$\alpha_{3,4}$ satellite have used the values presented in this work to fit theory with experiment. This work has found that fitting with the a priori shake-off probabilities as the values for the shake-off satellite intensities, results in a much poorer fit than when fitting the values after accounting for an Auger suppression factor—that is, when the K$\alpha_{3,4}$ satellite was fit with I(2s)/I(2p)=Pr(2s)/Pr(2p)=0.1787, the $\chi_{r}^{2}$ goodness-of-fit measure was consistently worse than when the fit was performed with the results from Table 10: I(2s)/I(2p)=0.0304.

## 11. Convergence Criteria

The MCDHF approach to calculating wavefunctions is an iterative process using the self-consistent field approach. The convergence of these iterations is defined and can be altered in the running of the GRASP-2018 software. The method of allowing for excitations into virtual orbitals by expanding the active set of CSFs, as mentioned in Section 3, results in different wavefunctions for what is nominally the same ground state. As more CSFs are used in the basis set to define the atomic state function, it becomes clear that the transition energies converge, as in Figure 1. No expansion of the active set—that is, no virtual orbital used in the basis set—uses 288 CSFs for defining the pre- and post-transition wavefunctions and results in a very broad, incorrect, eigenvalue spectra (first panel of Figure 1). The inclusion of virtual orbitals up to the 4f level, where one or two electrons may leave the canonical, or ground state, basis set and exist in either the 4p, 4d, or 4f orbital, allows for electron–electron correlation effects in the calculations. This expansion results in the eigenvalue spectrum in the second panel of Figure 1, where a better approximation of the Kα diagram transitions is found due to the discernible $\alpha_{1}$ and $\alpha_{2}$ structures. The inclusion of the 5s orbital increases the number of CSFs in the basis set from 2.4 $\times \;10^{6}$ to 3.1 $\times \;10^{6}$ and furthers the pattern of decreasing breadth and increasing structure. The final expansion presented in this work, from the 5s to the 5f, increases the size of the basis set to 10.5 $\times \;10^{6}$, substantially more than the difference between 4f and 5s; yet, the difference in the eigenvalue spectrum is imperceptible. This implies that a well-converged eigenvalue spectrum has been obtained.

The qualitative convergence in Figure 1 must be given a quantitative value. There are several measures of convergence that may be defined, each being the change in some calculated value as the expansion of the active set is applied. The values that are calculated for electron transitions are the energy eigenvalue spectra, intensities of the transition energies, and the Einstein coefficients for each eigenvalue.

**Figure 7:**
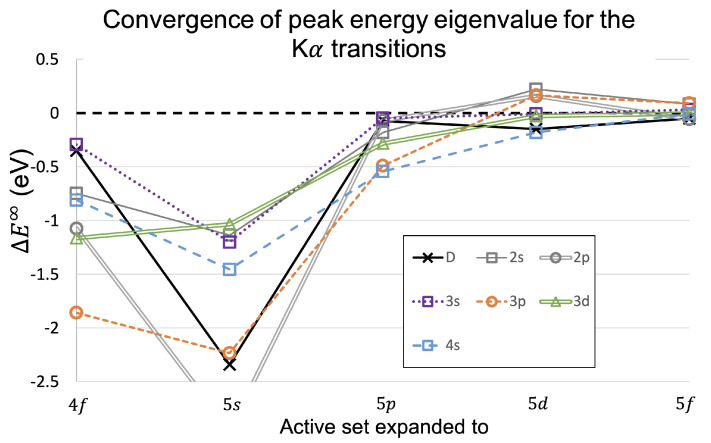
The values from Table 12 plotted. Here, we observe convergence of the peak energy eigenvalue of each set of transitions, the diagram, and each nl shake-off satellite.

One might consider how the most intense energy eigenvalue shifts as the active set is expanded, as applied by Nguyen et al. [14]. The values for the shift in peak energy eigenvalue, or $\Delta E^{\infty }$, are given by $\Delta E_{nl}^{\infty }=E_{nl}^{\infty }-E_{nl-}^{\infty }$, where the subscript nl− denotes the previous expansion subshell. The values for the peak energy eigenvalue are shown for the diagram and each nl satellite for the Kα transitions in Table 12 and Figure 7.

Figure 7 clearly shows that the peak energy eigenvalue can shift by up to 3 eV when the active set is expanded to include orbitals from the 4s to the 5s. Each peak eigenvalue was calculated to be lower in energy, as the active set was expanded from 4s to 4f, by between 0.35 eV and 1.87 eV. The shift down in energy was even greater as the 5s orbital was included in the active set, with the range in energy shifts from 1.04 eV to 2.95 eV. After the 5s level of calculation, the peak energies seemed to stabilise and not shift by more than 0.5 eV. This shows that a calculation without accounting for virtual orbitals, using only CSFs up to the 4s orbital in the basis set for the wavefunctions, can be incorrect by up to 4 eV.

The last expansion, from the 5d to 5f orbitals, shows that each peak value has converged to within 0.1 eV. This gives some indication on the error of our theoretically derived results, especially for the $E^{\infty }$ measure of the spectrum, since it only relies on the peak energy eigenvalue.

There are many thousands of transition energies, and we account for the convergence of all of these by taking a weighted mean or centre of mass (CoM) measure. The CoM of the eigenvalue spectrum is calculated through weighting each $E_{n}$ energy eigenvalue by its intensity, $g_{fn}$:

(3) $$E^{CoM}=\frac{\sum^{N}E_{n}g_{fn}}{\sum^{N}g_{fn}}$$

**Figure 8:**
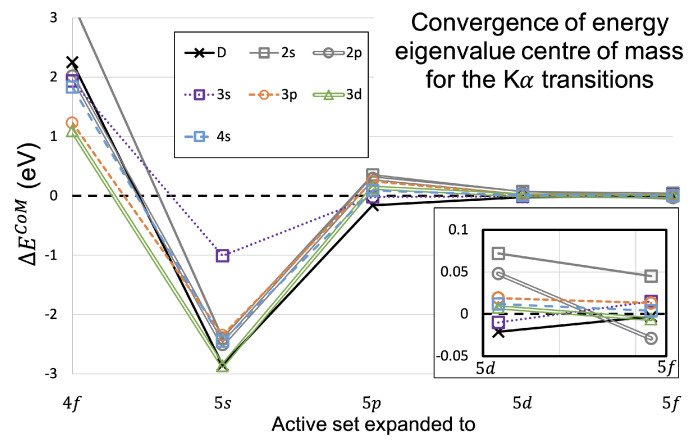
The change in the centre of mass energy eigenvalue for the Kα transitions as the active set is expanded from the 4s to the 5f level. The values are presented in Table 13. The box in the lower right expands the *y*-axis ($\Delta E^{CoM}$) for the last active set expansion, from level 5d to 5f.

Following this, the convergence of the CoM energy can be calculated from $\Delta E_{nl}^{CoM}=E_{nl}^{CoM}-E_{nl-}^{CoM}$ in the same way as $\Delta E_{nl}^{\infty }$ earlier. The use of a weighted mean, or CoM, convergence was established by Dean et al. in scandium due to the greater numbers of transition energies than copper [7]. The values for the peak energy eigenvalue are shown for the diagram and each nl satellite for the Kα transitions in Table 13 and Figure 8.

The most obvious difference between the pattern of convergence between the two measures, $\Delta E^{\infty }$ (Figure 7) and $\Delta E^{CoM}$ (Figure 8), is that the expansion from the 4s level to 4f creates a higher CoM energy but lower peak eigenvalue energy. The expansion from 4f to 5s shifts the CoM energy in the opposite direction to the initial expansion, which has the overall effect of having the initial 4s level of calculation be a reasonable guess for the true energy peak.

The last expansion, from the 5d to 5f levels, shows that the diagram and the n∈{3,4} shake-off satellite transitions, or the transitions responsible for the K$\alpha_{1,2}$ spectrum, change by less than 0.02 eV. The n=2 shake-off satellite transitions, those responsible for the K$\alpha_{3,4}$ satellite, shift by less than 0.05 eV at the last expansion. This gives some indication of how well-converged the calculation of the wavefunctions are and, therefore, how true the transition energies are. This level of convergence is comparable to the convergences found by Nguyen et al. for copper K$\alpha_{1,2}$ [13,14], Melia et al. for copper K$\alpha_{3,4}$ [8], and Dean et al. for scandium Kα and Kβ [7].

**Figure 9:**
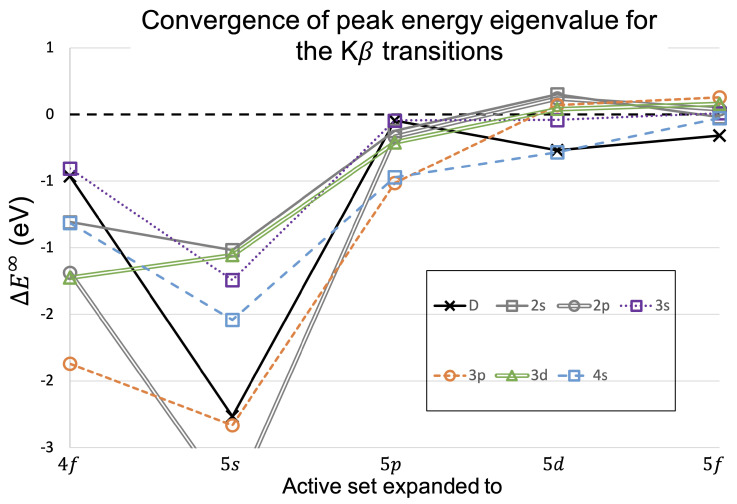
As in Figure 7 but for the Kβ transition energies, with the data presented in Table 14.

**Figure 10:**
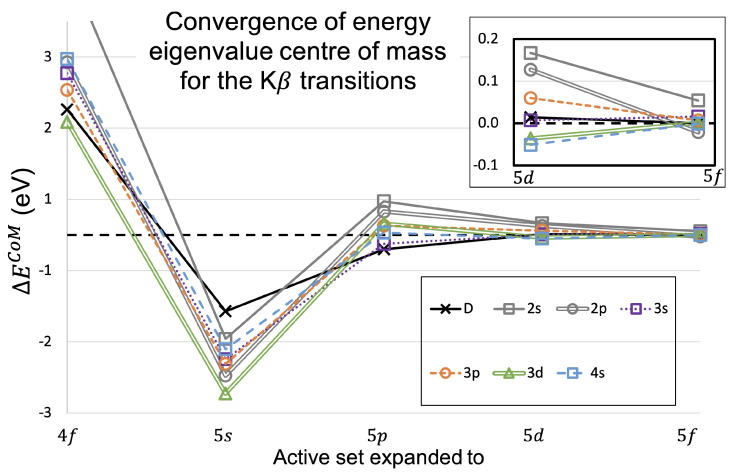
As in Figure 8 but for the Kβ transition energies, with the data presented in Table 15.

For the Kβ transitions, Table 14 and Figure 9 present the convergences for the peak energy eigenvalue. Table 15 and Figure 10 present the results for the CoM energy shifts for the Kβ transitions.

The convergence for the energies of Kβ transitions are very similar to the Kα transitions. The convergences are similar to the Kα trend, with the peak transition shifting down significantly for the first two expansions and the CoM value shifting first upward in energy and then downward. The convergence achieved on the last expansion for the CoM values shows the same feature as Kα, where the n=2 shake-off satellite transitions have converged far less well than the other sets of transitions. The convergence in Kβ is less convincing than in Kα, which follows a general trend of Kα calculations being more well-behaved than Kβ [6,7,8,13,14].

Along with the energy of each eigenvalue, the intensity of the *m*-th energy eigenvalue $g_{f,m}$ may also change as the active set is expanded, which provides another convergence criteria. Similar to the convergence of transition energies, we may compare the change in peak intensity of the nl level of expansion $\Delta g_{f,nl}^{\infty }$, which is less than 0.001% at each stage of the multiconfiguration expansion. If we take the relative change in the $g_{f}$ value, for the eigenvalue *m* between the expansion level nl and the previous nl−, and average over all of these in a set of transitions, we obtain the following formula:

(4) $$\Delta g_{f,nl}^{CoM}=\frac{\sum^{M}\left(\right|g_{f,m,nl}-g_{f,m,nl-}\left|\right)g_{f,m,nl}}{\sum^{M}g_{f,m,nl}}$$

This gives an insight into how all of the eigenvalue intensities change; yet, a similar conclusion is reached as with just the peak values. Between the 4s and 4f levels, there is some difference in the transition energies’ $g_{f}$ values observed but less than 1% in the mean difference and not more than 7% for any individual eigenvalue. After this first stage of the multiconfiguration expansion, expanding through levels 5s to 5f provides a change of no more than 0.01% for any individual eigenvalue at any step along the expansion. Hence, the $g_{f}$ values are not used as a definitive measure of convergence.

The last parameter to consider in convergence measures is how the Einstein coefficients *A* change. The Einstein coefficients are calculated via GRASP in two gauges, the length and velocity gauges, and give results denoted by $A^{L}$ and $A^{V}$, respectively. Ideally, the ratio of these two values is in unity, and this gives one measure of convergence, observing how $A^{L}/A^{V}$ changes as the active set is expanded. It is also important to observe that the value of $A_{m}^{L}$ for the *m*-th eigenvalue itself converges. As with the previous convergence measures, it is necessary to demonstrate the convergence of thousands of independent transition energies in an easy way, and this is conducted in two different ways: For the gauge ratio $A^{L}/A^{V}$, the value is calculated for each *m*-th eigenvalue at each nl level of expansion $A_{m,nl}^{L}/A_{m,nl}^{V}$, and the average value across every eigenvalue is taken, without weighting according to eigenvalue intensity. This is the only measure of convergence that is not defined by its change from the previous level, which allows the values at the 4s, or single configuration, level to be presented. These values for the Kα transitions are presented in Table 16 and for Kβ in Table 17.

These tables strongly support the convergence of the calculated wavefunctions. As the active set expands, the values for the gauge ratio trend towards unity. There are some exceptions; the Kβ4s shake-off satellite increases in the last two expansion steps and the Kβ 3*p* shake-off satellite increases in the last one step, with both being very small increases. The initial expansion from the single to multiconfiguration calculation is the most essential, providing much of the movement towards unity from the starting position.

Just observing the change in gauge ratio may overlook significant divergences in the Einstein coefficient that may be matched by similar divergences in both gauges; therefore, we consider the change in just the $A^{L}$ values. For the change in $A^{L}$ as the active set is expanded, the results are presented as the average fractional change in the value of $A^{L}$ for each eigenvalue or the average of $\left(A_{m,nl}^{L}-A_{m,nl-}^{L}\right)A_{m,nl}^{L}$. These results are shown in Table 18 for Kα and Table 19 for Kβ.

As before, these tables support the well-converged nature of the observables calculated and, therefore, the wavefunctions that they arise from. As with the gauge ratios presented, the largest jump is from single to multiconfiguration calculations. These values converge quickly, and rarely is there a change of more than one part in one-thousand between the 5d and 5f level of expansion. The Kα2p shake-off satellite is the only anomaly, which changes by a greater amount between the 5d and 5f levels than it did from the 5p and 5d.

Three independent convergence measures have now been presented for the change in energy, intensity, and Einstein coefficient. The energy convergence includes both the peak intensity eigenvalue and its convergence and the centre of mass convergence, and the Einstein coefficient includes both the change in the $A^{L}$ value alone and the $A^{L}/A^{V}$ ratio. In total, five measures of convergence have been reported. These values are calculated at the single configuration 4s level to the multiconfiguration inclusion of virtual orbitals in the basis set up to and including the 5f orbital, and the change between each level of expansion decreases each time. For each of the five measures of convergence, the trend towards zero is a key indicator that these calculations have converged.

The tables and figures presented thus far in this section give confidence that the wavefunctions calculated with the MCDHF method are well converged. With well-converged wavefunctions, credence is given to support the calculated observables. Specifically, Table 12, Table 13, Table 14 and Table 15 and their associated Figure 7, Figure 8, Figure 9 and Figure 10 enable an accurate claim to be made. For the Kα calculations, the CoM energy converged in the last step of the active set expansion to within 0.05 eV for every transition and to within 0.02 eV if we exclude the 2s and 2p values. Similarly, for Kβ, all transitions converged to obtain a CoM energy within 0.06 eV, and excluding the 2s and 2p values gives 0.02 eV. These values may be used as the uncertainty claims on the theoretically derived K$\alpha_{1}^{\infty }$, K$\alpha_{2}^{\infty }$, and K$\beta_{1,3}^{\infty }$ values. For the K$\alpha_{3,4}^{\infty }$ values, the convergence of the 2s and 2p transition energies should be used.

Transition energies are not the only observable calculated in this work. Uncertainty bounds may be obtained for the shake-off probability in the same way, by obtaining these values using the wavefunctions defined at each level of expansion of the active set. Shake-off probabilities are given as a percentage, and the change in this value from the 5f level calculation is given in Table 20. It is clear that the shake-off probabilities are highly robust during the expansion of the active set once the multiconfiguration states are included—that is, beyond the 4s level.

## 12. Conclusions

As theoretical calculations in the field of X-ray fluorescence improve, one hopes that the fit between theory and empirical data improves too. Where discrepancies remain, it is either due to incomplete understanding of the atomic physics processes, insufficient computing power to perform calculations of these processes, or phenomena that are not yet accounted for. This work advances the understanding of complex physical processes by providing calculations for the rates of competing processes to obtain satellite intensities that better model experimental data. We have demonstrated that using high-performance computers in conjunction with our GRASP software for atomic structure allows for well-converged wavefunctions for highly complex open atomic systems.

This work presents the full eigenvalue spectra for the Kα and Kβ transitions for manganese, including the canonical diagram lines, the nl shake-off satellite lines for nl∈{2s,2p,3s,3p,3d,4s}, and the valence-to-core [1s]→[3d] transition. Three different definitions of centroid energy are presented and compared with past theoretical and empirical studies. Shake-off probabilities are calculated, and Auger suppression is taken into account in order to present ab initio shake-off satellite intensities.

As the Auger process is accounted for in X-ray characteristic radiation, further studies will be able to probe even more subtle effects, which may require experimental data for state-of-the-art resolutions and precision. However, an accurate and precise account of the current models must be performed before these effects should be considered.

Due to the substantial number of possible angular momentum values, the number of eigenvalues is large and convergence is difficult. We have performed the active set approach to the 5f level of expansion, which results in a huge number of CSFs required in the basis set, anywhere between $10^{6}$ and $10^{7}$ depending on the transition. Several convergence criteria have been defined and presented in order to test the capabilities of the active set approach. Recent synchrotron data taken have been able to fit the K$\alpha_{3,4}$ spectra using the values from this work to a very good level of accuracy, $\chi_{reduced}^{2}=1.96$ [48]. Ongoing work suggests that similarly good fits will be obtained for K$\alpha_{1,2}$, K$\beta_{1,3}$, and K$\beta_{2,5}$.

## Figures and Tables

**Table 1:** The three results for the K$\alpha_{1}$ and K$\alpha_{2}$ energy depending on the definition of central tendency: $E^{0},E^{CoM}$, and $E^{\infty }$. The Lorentzian broadening used for $E^{0}$ are FWHM = 2.47 eV (K$\alpha_{1}$), 2.92 eV (K$\alpha_{2}$), following Hölzer et al. [42]. For $E^{\infty }$, only the strongest diagram transitions are used. For $E^{0}$ and $E^{\mathrm{CoM}}$, the ab initio satellite intensities from Table 9 are used to reconstruct the profile. Other theoretical and empirical results are presented for comparison. Uncertainties, where available, are presented in parentheses relative to the last digits of the value.

Result, eV	K$\alpha_{1}$	K$\alpha_{2}$
Theory		
This work, $E^{\infty }$	5899.981(52)	5888.945(61)
This work, $E^{0}$	5899.591	5888.595
This work, $E^{\mathrm{CoM}}$	5899.343(3)	5888.114(7)
Jonnard et al. [15]	5902.1	5891.2
Deslattes et al. [34]	5898.10(42)	5886.20(45)
Experiment		
Parratt [21]	5898.79	5887.73
Bearden [43]	5898.81(1)	5887.72(1)
Hölzer et al. [42]	5898.80(1)	5887.59(1)
Jonnard et al. [15]	5898.3(2) ^†^	5887.8(2) ^†^
Smale et al. [44]	5898.8010(84)	5887.6859(84)
Ito et al. [4]	5898.841(62)	5887.702(65)
Robledo et al. [47]	5898.56	5889.14
Tran et al. [45]	5900.3(1)	5889.1(1)
Mean experimental	5898.90	5888.06

^†^ The uncertainty is the result of the digitisation process.

**Table 2:** The values for the three definitions of central energy for the K$\alpha_{3}$, K$\alpha_{4}$, and combined K$\alpha_{3,4}$ eigenvalue spectra from this work. Previous experimental and theoretical work are included for comparison.

Result, eV	K$\alpha_{4}$	K$\alpha_{3}$	K$\alpha_{3,4}$
Theory			
This work, $E^{\infty }$	5926.760(62)	5931.989(55)	5931.989(55)
This work, $E^{0}$	5926.359	5931.578	5930.750
This work, $E^{\mathrm{CoM}}$	5925.782(21)	5930.611(29)	5929.814(29)
Mitra et al. [35]	-	-	5930.1
Experiment			
Parratt [21]	5924.72	5928.70	-
Mitra et al. [35]	-	-	5933(3)
Sier et al. [48]	5927.25	5930.92	5929.63

**Table 3:** The values for the three definitions of central energy for the K$\beta_{1}$, K$\beta_{3}$, and combined K$\beta_{1,3}$ eigenvalue spectra from this work. Previous experimental and theoretical work are included for comparison.

Result, eV	K$\beta_{1}$	K$\beta_{3}$	K$\beta_{1,3}$
Theory			
This work, $E^{\infty }$	6490.725	6476.803	6490.725
This work, $E^{0}$	6489.937	6477.532	6490.701
This work, $E^{\mathrm{CoM}}$	6489.622	6478.225	6485.69
Jonnard et al. [15]	-	-	6502.4
Deslattes et al. [34]	6492.7(1.0)	6485.39(96)	-
Mitra et al. [35]	-	-	6485.4(2)
Experiment			
Bearden, 1967 [43]	-	-	6490.50(1)
Hölzer et al. [42]	-	-	6490.18(1)
Jonnard et al. [15]	-	-	6490.2(2)
Mitra et al. [35]	-	-	6491(1)
Ito et al. [5]	6490.57(11)	6478.30(19)	6490.48(2)

**Table 4:** The theoretical results obtained in this work for the transitions relevant to the K$\beta_{2,5}$ satellite for manganese. The transitions include the 2s and 2p shake-off satellites—the column labelled [1s2l]→[3p2l], similar to K$\alpha_{3,4}$—and the dominant valence-to-core component [3d]→[1s]. Experimental data do not make this distinction, and only the peak of the profile is presented. Where possible, uncertainties are given.

Result, eV	[1s]→[3d]	[1s2l]→[3p2l]
Theory		
This work, $E^{\infty }$	6533.179	6550.614
This work, $E^{0}$	6533.182	6550.635
This work, $E^{\mathrm{CoM}}$	6534.430	6550.322
Deslattes et al., 2003 [34]	6538.54(51)	-
Mitra et al., 2008 [35]	6549.9	-
Experiment	K$\beta_{2,5}$	
Jonnard et al., 2002 [15]	6536.1(2)	
Mitra et al., 2008 [35]	6550(3)	
Ito et al., 2018 [5]	6534.54(18) *	

* Refers to the K$\beta_{2,5}$ satellite as just K$\beta_{5}$.

**Table 5:** Shake-off probabilities as calculated by this work, Kochur et al. [23], and Mukoyama and Taniguchi [22]. The normalised probabilities for the shake-off events and the diagram sum to 100%.

nl Shake-Off Probability (%)	2*s*	2*p*	3*s*	3*p*	3*d*	4*s*	No Shake
This work	0.168	0.940	0.842	4.240	10.253	15.042	68.515
Mukuyama & Taniguchi [22]	0.134	0.669	0.519	4.003	9.478	11.062	74.135
Kochur et al. [23]	0.26	1.17	1.01	4.56	10.95	14.08	67.97
Jonnard et al. [15]	0.27	0.71	1.46	4.56	-	-	-

**Table 6:** Transition rates of the competing processing possible after an n=2 shake-off event, the [1s2s] and [1s2p] hole states. The next possible single shake-off states are presented in Table 7. These values sum to yield the Auger suppression, with the relative weights between the total Auger rate and radiative rate for each initial hole state presented in the second row of the tree diagram in Figure 6.

Initial Hole	Final Hole(s)	Type	Name	Rate (eV/ℏ)
[1s2s]	[2p2s]	Radiative	Kα 2s sat.	0.141
	[3p2s]	Radiative	Kβ 2s sat.	0.024
	[1s2p3s]	Auger	$L_{1}$ $L_{2,3}$ $M_{1}$	1.248
	[1s2p3s]	Auger	$L_{1}$ $L_{2,3}$ $M_{2,3}$	2.246
	[1s2p3d]	Auger	$L_{1}$ $L_{2,3}$ $M_{4,5}$	2.483
	[1s2p4s]	Auger	$L_{1}$ $L_{2,3}$ $N_{1}$	0.664
	[1s3s3s]	Auger	$L_{1}$ $M_{1}$ $M_{1}$	0.008
	[1s3s3p]	Auger	$L_{1}$ $M_{1}$ $M_{2,3}$	0.132
	[1s3s3d]	Auger	$L_{1}$ $M_{1}$ $M_{4,5}$	0.492
	[1s3p3p]	Auger	$L_{1}$ $M_{2,3}$ $M_{2,3}$	1.598
	[1s3p3d]	Auger	$L_{1}$ $M_{2,3}$ $M_{4,5}$	2.874
	[1s3d3d]	Auger	$L_{1}$ $M_{4,5}$ $M_{4,5}$	1.701
	[1s3l4s]	Auger	Σ ${\mathrm{LMN}}_{1}$	<0.01
Total $L_{1}$(2s) Auger rate: 12.855				
[1s2p]	[2p2p]	Radiative	Kα 2p sat.	0.241
	[3p2p]	Radiative	Kβ 2p sat.	0.039
	[1s3s3s]	Auger	$L_{2,3}$ $M_{1}$ $M_{1}$	0.022
	[1s3s3p]	Auger	$L_{2,3}$ $M_{1}$ $M_{2,3}$	0.107
	[1s3s3d]	Auger	L$L_{2,3}$$M_{1}$$M_{4,5}$	0.248
	[1s3p3p]	Auger	L$L_{2,3}$$M_{2,3}$$M_{2,3}$	1.323
	[1s3p3d]	Auger	$L_{2,3}$ $M_{2,3}$ $M_{4,5}$	0.896
	[1s3d3d]	Auger	$L_{2,3}$ $M_{4,5}$ $M_{4,5}$	1.155
	[1s3l4s]	Auger	Σ ${\mathrm{LMN}}_{1}$	<0.01
Total $L_{2,3}$ (2p) Auger rate: 3.763				

**Table 7:** Transition rates of the competing processing possible after the n=3 and 4s shake-off satellites. These values contribute to the Auger suppression, with the relative weights between the total Auger rate and radiative rate for each initial hole state presented in the second row of the tree diagram in Figure 6.

Initial Hole	Final Hole(s)	Type	Name	Rate (eV/ℏ)
[1s3s]	[2p3s]	Radiative	Kα 3s sat.	0.174
	[3p3s]	Radiative	Kβ 3s sat.	0.020
	[1s3p3p]	Auger	$M_{1}$ $M_{2,3}$ $M_{2,3}$	0.018
	[1s3p3d]	Auger	$M_{1}$ $M_{2,3}$ $M_{4,5}$	0.032
	[1s3d3d]	Auger	$M_{1}$ $M_{4,5}$ $M_{4,5}$	0.034
	[1s3p4s]	Auger	$M_{1}$ $M_{2,3}$ $N_{1}$	0.048
	[1s3d4s]	Auger	$M_{1}$ $M_{4,5}$ $N_{1}$	0.037
Total $M_{1}$ (3s) Auger rate: 0.197				
[1s3p]	[2p3p]	Radiative	Kα 3p sat.	0.116
	[3p3p]	Radiative	Kβ 3p sat.	0.018
	[1s3p3p]	Auger	$M_{2,3}$ $M_{2,3}$ $M_{2,3}$	0.00
	[1s3d3d]	Auger	$M_{2,3}$ $M_{4,5}$ $M_{4,5}$	<0.01
	[1s3d4s]	Auger	$M_{4,5}$ $M_{4,5}$ $M_{4,5}$	0.00
Total $M_{2,3}$ (3p) Auger rate: <0.01				
[1s3d]	[2p3d]	Radiative	Kα 3d sat.	0.149
	[3p3d]	Radiative	Kβ 3d sat.	0.027
	[1s3d3d]	Auger	$M_{4,5}$ $M_{4,5}$ $M_{4,5}$	0.00
	[1s3d4s]	Auger	$M_{4,5}$ $M_{4,5}$ $N_{1}$	<0.01
Total $M_{4,5}$ (3d) Auger rate: <0.01				

**Table 8:** The calculated satellite and diagram intensities as a percentage of the total Kα from using the Auger suppression factors along with the shake-off probabilities. The nl atomic orbital listed represents the nl shake-off satellite.

Kα Ab Initio	Diagram	2*s*	2*p*	3*s*	3*p*	3*d*	4*s*
Intensities (%)	68.515	0.0018	0.0592	0.415	4.452	10.764	15.793

**Table 9:** Values from Table 8 renormalised for the K$\alpha_{1,2}$ spectrum in isolation.

K$\alpha_{1,2}$ Ab Initio	Diagram	3*s*	3*p*	3*d*	4*s*
Intensities (%)	68.515	0.415	4.459	10.786	15.825

**Table 10:** Values from Table 8 renormalised for the K$\alpha_{3,4}$ spectrum in isolation.

K$\alpha_{3,4}$ Ab Initio	2*s*	2*p*
Intensities (%)	2.95%	97.05%

**Table 11:** The calculated satellite and diagram intensities as a percentage of the total Kβ using the Auger suppression factors along with the shake-off probabilities. The nl atomic orbital listed represents the nl shake-off satellite.

Kβ Ab Initio	Diagram	2*s*	2*p*	3*s*	3*p*	3*d*	4*s*
Intensities (%)	69.81	0.00035	0.0096	0.088	4.320	10.447	15.326

**Table 12:** The change in the peak energy eigenvalue—the eigenvalue with the greatest intensity, or $g_{f}$ value, for the diagram and each of the nl shake-off satellite (sat.). The value is given as the current level, taking the previous level centre of mass energy, except for the 4f level, which is taken as the difference from the 4s. These results are presented in Figure 7.

Kα $\Delta E^{\infty }$ (eV)					
Transition	4f	5s	5p	5d	5f
Diagram	−0.350	−2.341	−0.073	−0.150	−0.052
2s sat.	−0.749	−1.137	−0.182	0.220	0.082
2p sat.	−1.074	−2.950	−0.070	0.159	−0.055
3s sat.	−0.295	−1.202	−0.049	−0.010	0.031
3p sat.	−1.859	−2.236	−0.492	0.165	0.091
3d sat.	−1.164	−1.036	−0.283	−0.028	−0.005
4s sat.	−0.811	−1.455	−0.546	−0.181	−0.009

**Table 13:** The change in the centre of mass energy eigenvalue for the Kα transitions as the active set is expanded from the 4s to the 5f level, for the diagram and each of the nl shake-off satellites (sat.). The value is given as the current level, taking the previous level centre of mass energy, except for the 4f level, which is taken as the difference from the 4s. These results are presented in Figure 8.

Kα $\Delta E^{\mathrm{CoM}}$ (eV)					
Transition	4f	5s	5p	5d	5f
Diagram	2.251	−2.849	−0.159	−0.021	−0.003
2s sat.	3.262	−2.396	0.353	0.072	0.045
2p sat.	2.018	−2.503	0.295	0.048	−0.029
3s sat.	1.938	−1.009	−0.019	−0.010	0.015
3p sat.	1.230	−2.349	0.248	0.019	0.013
3d sat.	1.096	−2.862	0.138	0.008	−0.006
4s sat.	1.834	−2.448	0.089	0.012	0.004

**Table 14:** The convergence of the peak energy eigenvalue in the Kβ transitions, as shown in Table 12.

Kα $\Delta E^{\infty }$ (eV)					
Transition	4f	5s	5p	5d	5f
Diagram	−0.464	−2.264	−0.046	−0.268	−0.158
2s sat.	−0.808	−1.018	−0.123	0.150	−0.021
2p sat.	−1.188	−2.916	−0.168	0.121	0.039
3s sat.	−0.406	−1.243	−0.046	−0.042	0.009
3p sat.	−1.870	−2.333	−0.514	0.071	0.128
3d sat.	−1.225	−1.056	−0.210	0.040	0.079
4s sat.	−0.814	−1.542	−0.472	−0.284	−0.027

**Table 15:** The convergence of the energy eigenvalue CoM in the Kβ transitions, as shown in Table 13.

Kα $\Delta E^{\mathrm{CoM}}$ (eV)					
Transition	4f	5s	5p	5d	5f
Diagram	1.762	−1.072	−0.201	0.014	0.000
2s sat.	3.805	−1.458	0.473	0.167	0.054
2p sat.	2.423	−1.975	0.330	0.127	−0.021
3s sat.	2.270	−1.742	−0.125	0.007	0.016
3p sat.	2.039	−1.809	0.130	0.060	0.007
3d sat.	1.588	−2.228	0.158	−0.036	0.001
4s sat.	2.471	−1.604	0.033	−0.051	−0.001

**Table 16:** The mean value of the gauge ratio $A^{L}/A^{V}$ for each of the Kα transitions.

	Kα ${\left(A^{L}/A^{V}\right)}_{\mathrm{nl}}$					
Transition	4s	4f	5s	5p	5d	5f
Diagram	1.117	1.045	1.018	1.015	1.015	1.015
2s sat.	1.105	1.053	1.027	1.022	1.022	1.022
2p sat.	1.179	1.056	1.030	1.024	1.023	1.023
3s sat.	1.085	1.050	1.025	1.016	1.017	1.015
3p sat.	1.175	1.052	1.017	1.015	1.013	1.012
3d sat.	1.162	1.048	1.013	1.014	1.014	1.014
4s sat.	1.207	1.051	1.026	1.022	1.018	1.018

**Table 17:** The mean value of the gauge ratio $A^{L}/A^{V}$ for each of the Kβ transitions.

	Kβ ${\left(A^{L}/A^{V}\right)}_{\mathrm{nl}}$					
Transition	4s	4f	5s	5p	5d	5f
Diagram	1.142	1.033	1.021	1.021	1.021	1.021
2s sat.	1.189	1.074	1.043	1.040	1.040	1.039
2p sat.	1.183	1.065	1.030	1.029	1.029	1.028
3s sat.	1.133	1.047	1.025	1.026	1.027	1.027
3p sat.	1.119	1.088	1.037	1.035	1.033	1.035
3d sat.	1.080	1.070	1.034	1.032	1.030	1.030
4s sat.	1.125	1.050	1.024	1.022	1.023	1.024

**Table 18:** The fractional convergence of the Einstein coefficients in the length gauge $A^{L}$ for each of the Kα transitions as the active set is expanded.

Kα $\Delta A^{L}$					
Transition	4f	5s	5p	5d	5f
Diagram	0.078	0.044	0.003	0.002	0.000
2s sat.	0.093	0.084	0.003	0.001	0.001
2p sat.	0.067	0.053	0.002	0.002	0.003
3s sat.	0.082	0.023	0.002	0.001	0.001
3p sat.	0.072	0.034	0.003	0.001	0.000
3d sat.	0.058	0.014	0.003	0.002	0.001
4s sat.	0.089	0.054	0.003	0.002	0.000

**Table 19:** The fractional convergence of the Einstein coefficients in the length gauge $A^{L}$ for each of the Kβ transitions as the active set is expanded.

Kβ $\Delta A^{L}$					
Transition	4f	5s	5p	5d	5f
Diagram	0.085	0.053	0.005	0.000	0.000
2s sat.	0.110	0.076	0.008	0.002	0.002
2p sat.	0.130	0.097	0.009	0.003	0.000
3s sat.	0.090	0.061	0.002	0.002	0.001
3p sat.	0.120	0.085	0.006	0.000	0.000
3d sat.	0.105	0.070	0.002	0.000	0.000
4s sat.	0.140	0.095	0.008	0.006	0.001

**Table 20:** The shake-off probability has been calculated at each level of expansion of the active set, and the difference between the nl level and the 5f level are presented, taken as the 5f level subtract the nl level. The values in the 5f column are the final values and are also given in Table 5. The values in the 5p column do not sum to zero due to rounding errors beyond the third decimal.

Shake-Off Probability (%) Relative to 5f Level of Calculation						
nl Shake-Off	4s	4f	5s	5p	5d	5f (%)
No shake	4.505	0.104	0.286	0.004	0.000	68.515
4s	−1.453	−0.032	−0.095	−0.002	0.000	15.042
3d	−1.650	−0.011	−0.017	−0.001	0.000	10.253
3p	−0.886	−0.019	−0.076	0.000	0.000	4.240
3s	−0.380	−0.012	−0.053	0.000	0.000	0.842
2p	−0.105	−0.020	−0.041	0.000	0.000	0.940
2s	−0.031	−0.010	−0.004	0.000	0.000	0.168

## Data Availability

We have made the GRASP output files available in Supplemental Information.

## Supplementary Materials

The following supporting information can be downloaded at: https://www.mdpi.com/1420-3049/29/17/4199/s1, The Supplementary Materials include the .ct files containing the transition energies, line strengths, and Einstein coeffecients for every set of transitions. The set of transitions is denoted as “Mn_Ka_D-5s” for the manganese Kα diagram set of transitions expanded to the 5s orbital. Substitute “Ka” with “Kb” for the Kβ transitions, and replace “D” with “nl” for the nl shake-off satellite set of transitions. Also included are binary files for each set of transitions to easily read in an array of transitions energies and their relative intensities.

## Abbreviations

The following abbreviations are used in this manuscript:

FWHM	Full-width at half-maximum
GRASP	General Relativistic Atomic Structure Package
MCDHF	Multiconfiguration Dirac–Hartree–Fock
QED	Quantum electrodynamics
RATIP	Relativistic Atomic Transitions and Ionisation Properties
